# The effect of trifluridine/tipiracil for patients with heavily pretreated metastatic gastric cancer: A protocol for systematic review and meta-analysis

**DOI:** 10.1097/MD.0000000000024110

**Published:** 2021-01-15

**Authors:** Xiaoyan He, Tianyao Zhang, Lijuan Wu, Yongcan Wu, Xin Zhou

**Affiliations:** a College of Basic Medicine; b College of Clinical Medicine, Chengdu University of Traditional Chinese Medicine, Chengdu, Sichuan; c Department of Endocrinology, Chongqing Traditional Chinese Medicine Hospital, Chongqing, China.

**Keywords:** metastatic gastric cancer, protocol, systematic review, trifluridine/tipiracil

## Abstract

**Background:**

Gastric cancer is a global health problem with high incidence rate and mortality rate. Due to the limitations of traditional chemotherapy drugs, such as patient intolerance, low efficacies and serious adverse effects, trifluridine/tipiracil has been considered to be a promising treatment for patients with heavily pretreated metastatic gastric cancer. However, the relevant systematic review has not been occurring. The presentation of this protocol is to scientifically evaluate the efficacy and safety of trifluridine/tipiracil in patients with highly pretreated metastatic gastric cancer.

**Methods:**

The protocol followed Preferred Reporting Items for Systematic Reviews and Meta-Analyses Protocols. We will systematically search MEDLINE, PubMed, Embase, Cochrane Library, Web of Science, China National Knowledge Infrastructure Database (CNKI), VIP Chinese Science and Technology Periodical Database (VIP), Wan Fang Database up to November 1, 2020 to identify published articles. Using the Cochrane risk assessment tool to assess the methodological quality of the RCTs, and all included studies will be analyzed according to the criteria in the Cochrane Handbook. Review Manager 5.3 software will be used for literature quality evaluation and data analysis.

**Results:**

Objective to evaluate the efficacy and safety of trifluridine/tipiracil in patients with heavily pretreated metastatic gastric cancer by analyzing the eligible data extracted under limited conditions.

**Conclusion:**

This study provides clear evidence to evaluate the effectiveness and safety of trifluridine/tipiracil for patients with highly pretreated metastatic gastric cancer, and the findings will also be published in a peer-reviewed journal.

**Ethics and dissemination:**

No ethical statement will be required for this study because there is no direct involvement of human. This review will be published in a peer-reviewed journal as a conference report or an article.

**Registration:**

OSF registration number: DOI 10.17605/OSF.IO/6MF5U.

## 1 Introduction

Gastric cancer (GC), a global health problem, is the fifth most common cancer in the world, and the third most common cause of cancer-related death, according to the GLOBOCAN 2018 data.^[1,2]^ More than 1 million people were diagnosed with GC and caused 783,000 deaths every year.^[2,3]^ The 5-year survival rate for GC is 31% in the United States (US) and 19% in the United Kingdom. In Europe, the average 5-year survival rate is 26%.^[2]^ Most patients with GC are advanced or metastatic disease, and palliative chemotherapy is the mainstay method.^[4]^ Multimodal treatment including surgical resection is the only potential treatment standard for resectable T2-T4 and/or lymph node-positive diseases. However, 40% to 60% of resection patients eventually relapse, and two-thirds of patients still have advanced disease that can no longer be operated on.^[5]^ Previously recommended first-line and second-line treatments have low tolerance and efficacy, the prognosis of patients with advanced GC is very poor, and the median overall survival (OS) time is 10 to 12 months.^[4,6]^ Therefore, the emergence of new treatment methods and drugs is of great significance.

Trifluridine/tipiracil (Lonsurf; TAS-102) has been approved by the national health authorities of the US, Japan and Europe for the treatment of patients with metastatic colorectal cancer who have received or are unable to receive other available treatments, including chemotherapy with fluoropyrimidine, oxaliplatin, irinotecan, and antivascular endothelial growth factor biologic products, anti-epidermal growth factor receptor monoclonal antibodies, if rat sarcoma viral oncogene homologue wild type.^[6,7]^ As trifluridine/tipiracil has been shown to be effective for heavily pretreated advanced GC, it has been approved for use in the US in February 2019, in Japan in August 2019 and in the European Union in September 2019.^[8]^

Trifluridine/tipiracil consists of thymidine nucleoside analog (trifluoropyridine) and thymidine phosphorylase (TP) inhibitor (tipiracil hydrochloride) in a molar ratio of 1:0.5.^[6,7]^ Trifluridine, a nucleoside analogue based on thymidine, is an active cytotoxic component of trifluridine/tipiracil. It can be metabolized into a triphosphate metabolite and then incorporated into deoxyribonucleic acid (DNA) to inhibit DNA synthesis and function, furthermore, its antitumor activity is mainly based on its incorporation into DNA.^[7,8]^ Trifluridine also inhibits thymidylate synthase, which is used to synthesize deoxycytidine monophosphate for DNA synthesis. However, this inhibition of thymidylate synthase plays a smaller role in the biological activity of the molecule.^[6,7]^ As a TP inhibitor, tipiracil can inhibit the degradation of trifluridine through TP. When trifluridine was administered alone, it was rapidly metabolized into inactive forms by TP in liver and gastrointestinal tract.^[9]^ If tipiracil was added to the formulation, the bioavailability and cytotoxicity of trifluridine can be improved by inhibiting the degradation of trifluridine.^[6,10]^ Clinical research shown that patients with advanced GC have well tolerance to trifluridine/tipiracil, and no treatment-related deaths.^[11]^ In addition, in the late-line therapy of patients who failed all available treatments (including fluoropyrimidines), the median OS (primary end point) of trifluridine/tipiracil was continuously improved compared with placebo.^[6]^ Concomitantly, clinical researches on the treatment of patients with heavily pretreated metastatic gastric cancer by trifluridine/tipiracil are also increasing. Therefore, its clinical efficacy and safety are worth exploring.

## 2 Methods

### 2.1 Study registration

This study has been registered in OSF, registration number: DOI 10.17605/OSF.IO/6MF5U. And the protocol of this meta-analysis is based on the preferred reporting items for systematic reviews and meta-analyses protocols (PRISMA-P) guidance.^[12]^

### 2.2 Database and search strategy

We will systematically search MEDLINE, PubMed, Embase, Cochrane Library, Web of Science, China National Knowledge Infrastructure Database (CNKI), VIP Chinese Science and Technology Periodical Database (VIP), Wan Fang Database up to 1 November 2020 to identify published articles. Clinical studies with “trifluridine and tipiracil”, “TAS-102” monotherapy in series of patients evaluable for effectiveness will be included. The articles will not be limited in language. We will exclude papers reporting phases I, II, or III trials, case reports and series with less than 20 patients, and publications only in abstract form. To locate additional relevant publications, we will search the references list from each relevant article or the retrieved articles manually. Furthermore, we will contact experts in the field to gather information on the ongoing and unpublished studies. Retrieve formula is listed in Table 1.

**Table 1:** The search strategy in Pubmed.

Search	Search term
#1.	randomized controlled trial [Title/Abstract]
#2.	controlled clinical trial [Title/Abstract].
#3.	randomized [Title/Abstract]
#4.	randomly [Title/Abstract]
#5.	RCT [Title/Abstract]
#6.	#1 OR #2 OR #3 OR #4 OR #5
#7.	pretreated metastatic gastric cancer [MESH]
#8.	heavily pretreated metastatic gastric cancer [Title/Abstract]
#9.	metastatic gastric cancer [Title/Abstract]
#10.	gastric cancer [Title/Abstract]
#11.	#7 OR #8 OR #9 OR #10
#12.	Trifluridine/tipiracil [MESH]
#13.	Lonsurf [Title/Abstract]
#14.	TAS102 [Title/Abstract]
#15.	#12 OR #13 OR #14
#16.	#6AND #11 AND #15

### 2.3 Inclusion criteria

#### 2.3.1 Types of studies

Original article with randomized controlled trials (RCTs), according to Cochrane Collaboration's RCT criteria, all references to the words “random sequence” are regarded as RCTs, regardless of single-blind, double-blind or non-blind. Languages are not limited to English.

#### 2.3.2 Participants

Participants aged between 18 to 75 years old will be included. Patients with heavily pretreated metastatic gastric cancer (histologically confirmed, non-resectable, metastatic gastric adeno carcinoma or/and adenocarcinoma of the gastroesophageal junction) as defined by the American Joint Committee on Cancer staging classification (7th edition).^[13]^ They had previously received 2 or more standard-of-care regimens for advanced disease and had experienced radiological disease progression within 3 months of the last dose of the last previous therapy or were untolerable their last previous therapy.

#### 2.3.3 Intervention

Interventions in the experimental group are “trifluridine and tipiracil” monotherapy, while the control group are placebo. Patients received oral trifluridine/tipiracil 35 mg/m^2^ twice daily plus best supportive care or placebo twice a day plus best supportive care on days 1–5 and days 8–12 of each 28-day treatment cycle, and the course of treatment is more than or equal to 2 cycles.

#### 2.3.4 Outcome measures

##### 2.3.4.1 Primary outcome

The overall survival (OS) and progression-free survival (PFS) have been analyzed as the primary outcome. The time of OS was from randomization to death, and PFS was from randomization to investigation or assessment of radiation disease progression or death from any cause.

##### 2.3.4.2 Secondary outcome

1. ORR (objective response rate; randomization start plus two-time or four-time cancer evaluations): the proportion of patients with a complete response or a partial response according to RECIST v1.1.^[14]^
2. DCR (disease control rate; randomization start plus two-time or four-time cancer evaluations): the proportion of patients with a complete response, a partial response, or stable disease.
3. ECOG (Eastern cooperative oncology group) performance status (https://ecog-acrin.org/resources/ecog-performance-status). The record time from randomization until the first date on which an ECOG performance status score of 2 or higher.
4. Adverse events will be graded according to the US National Cancer Institute's Common Terminology Criteria for Adverse Events (CTCAE; version 4.0).^[15]^ And the record time at the beginning of drug therapy.

### 2.4 Exclusion criteria

We will not include studies as follows:

1. Articles with unclear tables, figures, inclusion and exclusion criteria.
2. Studies which do not meet the above mentioned inclusion criteria.
3. Duplicate publications, case series, reviews, observation study, animal research and pharmacological experiments.
4. Studies in which the treatment has been combined with another therapy.
5. Case reports and series with less than 20 patients.
6. Intervention duration less than 2 cycles.

### 2.5 Study selection and data extraction

We will refer to the Cochrane collaborative network system evaluator handbook.^[16]^ Using Note Express document management software (version 3.2; Beijing Aegean Software Company, Beijing, China; https://en.freedownloadmanager.org) to import the search results. Eliminating duplicate and irrelevant articles by reading the article title and abstract.

Two authors (XY He and TY Zhang) will independently extract data through the predesigned form, the relevant items as following: the name of the first author, publication year, patient demographics (i.e., age, number, and drug administered), median treatment duration, median follow up (months), study design (i.e., the type of blinding, the type of control, the methods for randomization), survival outcomes expressed as hazard ratios (HRs) for OS and PFS, additional outcomes and the number of patients who came from the intervention group and the control group. Any disagreement will be resolved through discussion with a third reviewer. All articles included are judged by the third reviewer (Xin Zhou). If the consensus still cannot be reached, the dispute shall be settled by contacting the original author for original data.

**Figure 1:**
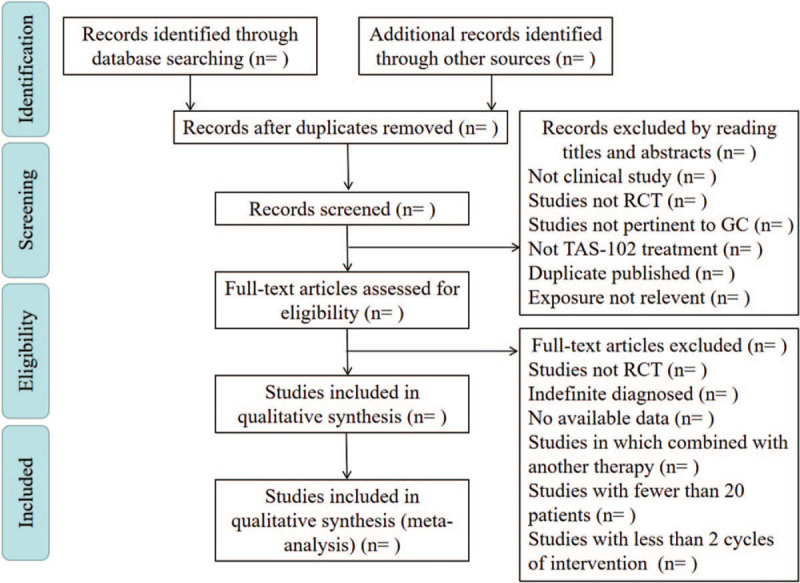
Preferred Reporting Items for Systematic Reviews and Meta-Analyses flow chart of study selection process.

The whole selection process will be presented in a PRISMA flow diagram (Fig. 1). Quantitative data for meta-analysis will be extracted on a separate extraction sheet. Review Manager 5.3 software will be used for literature quality evaluation, data synthesis and analysis.

### 2.6 Assessment of methodologic quality

We will use the Cochrane risk assessment tool to assess the risk of bias,^[17]^ including double blinding and concealment of assignments, adequacy of allocation sequence generation, incomplete outcome dates, selective outcome reporting, follow-up, and other biases. It is classified as “yes” if adequate, otherwise, it is classified as “no”. In addition, it is classified as “unclear” if the author does not well describe in such a way that its adequacy is describable.

Two researchers (LJ Wu and YC Wu) will independently evaluate the risk of bias for each included study. The tool has 7 components: a. adequate sequence generation; b. concealment of assignment; c. blind method (participants and personnel); d. blind method (outcome evaluator); e. incomplete result data processed (ITT analysis); f. selective reporting; g. other potential threats to effectiveness. Each of these indicators is divided into high bias risk, low bias risk, and ambiguity. We will use “H” to indicate high bias risk, “L” to indicate low bias risk, and “U” to indicate the bias risk is unclear. And we will use a scale from 0 to 7. Studies will be scored, a score of 1 or 2 being considered to be of low quality, while trials with a score of 3–5 are considered to be of high quality. Studies meeting at least 3 criteria will be included in the final analysis. All researchers resolved their disagreements through discussion. If necessary, we will contact the study authors to inquire missing information. Furthermore, trials of high risk of bias should be considering execute sensitive analysis.

### 2.7 Data synthesis and statistical analysis

The statistical analyses will be performed by Cochrane Collaboration Review Manager V.5.3.5.5, for all the statistical analyses, a value of *P* < .05 will be regarded as statistically significant, and all tests will be two-sided. In addition, for the time-to-event variables, including OS, PFS, HRs with 95% confidence intervals (CI) will be calculated for each study. For the dichotomous variables, risk ratios (RRs) with 95% CIs will be calculated for each study; continuous outcomes weighted will be adopted for mean difference.

Homogeneity of risk estimates between studies is assessed using the *I*^2^ statistic and the Cochran Q statistic. *I*^2^ < 50% or *P* > .10 indicated no statistical heterogeneity, the fixed-effect model (Mantel-Haenszel method) will be used to analyze. Otherwise, random-effects model (DerSimonian-Laird method) will be calculated. If the heterogeneity between studies remains high after subgroup analysis, sensitivity analysis will be performed. After excluding the low-quality study, a meta-analysis is conducted again to compare the new combined results with the previous results. If there is no significant change between the 2 results, the sensitivity will be very low. By contrary, there is a big difference between the 2 results, or even get the opposite conclusion, the sensitivity is high. However, if heterogeneity still remains high detected, recognized methods that based on clinical parameters will be used to explore it.

### 2.8 Subgroup analysis

Subgroup analysis will be based on the possible factors that may lead to heterogeneity. We will conduct subgroup analysis according to race, age, tumor location (gastric versus gastro-oesophageal junction), gender and Eastern Cooperative Oncology Group (ECOG) performance status (1 vs 0) (https://ecog-acrin.org/resources/ecog-performance-status). If quantitative synthesis is not appropriate, we will conduct a narrative synthesis.

### 2.9 Publication bias

Publication bias will be explored by funnel plot analysis if a sufficient number of studies (n > 10) are available and quantitative test of publication bias by Begg method. On the contrary, Egger test will be applied.

## 3 Discussion

At present, there is no screening method to detect GC, and patients with early GC usually have no symptoms, so the early diagnosis rate of GC is low, and more than 70% of patients with advanced GC.^[18]^ For patients with unresectable advanced GC, systemic chemotherapy is a standard treatment.^[19]^ However, resistance to chemotherapeutic agents often leads to treatment failure,^[20]^ once resistance appears, treatment programs will become limited.^[21]^ Therefore, more optimized chemotherapy agents and therapy methods will be required.

Clinical studies have shown that trifluridine/tipiracil has a positive efficacy on patients with advanced GC and its toxicity is acceptable,^[10]^ moreover, trifluridine/tipiracil monotherapy was beneficial to the median PFS and OS values of patients with advanced GC,^[10,19,21]^ and it has well tolerance.^[18]^ Trifluridine has 2 antitumor mechanisms. It not only inhibits thymidylate synthase, which is similar to fluorouracil, but also produce double-stranded DNA breaks by incorporating the triphosphate form into DNA.^[21]^ Tipiracil can improve the pharmacokinetics of trifluridine when used in combination with trifluridine, thereby increasing the drug concentration and antitumor activity.^[22]^ Although trifluridine is classified as fluoropyrimidine, its main mechanism of action is different from that of traditional fluoropyrimidines such as 5-FU, allowing for make trifluridine overcome 5-FU resistance.^[8,20,23,24]^ As a relatively new oral fluoropyrimidine therapy, trifluridine/tipiracil is a useful treatment option in addition to second-line treatment for patients who have made progress in previous treatment or have limited therapy options and poor prognosis. However, there are many adverse events of trifluridine/tipiracil include myelosuppression, haematological adverse events such as leucopenia, thrombocytopenia, neutropenia, anaemia, gastrointestinal events such as nausea, vomiting, diarrhoea and other symptoms.^[25,26]^ In addition, due to the higher incidence of grade 3 or 4 hyperbilirubinaemia in patients with moderate to severe hepatic insufficiency, the use of trifluridine/tipiracil is not recommended. It is also not recommended for patients with severe renal insufficiency or end-stage renal disease.^[20,25]^ These adverse events cannot be ignored, it is necessary to evaluate its effectiveness and safety based on a large number of reliable clinical trial results in order to better guide clinical medication.

In conclusion, we will conduct follow-up systematic research based on the existing RCTs data of trifluridine/tipiracil treatment of heavily pretreated metastatic GC, and we will review it according to the process of determining the research criteria, data retrieval, data extraction and data analysis. This is the first time to evaluate the efficacy and safety of trifluridine/tipiracil in the treatment of heavily pretreated metastatic GC, which is innovative. At the same time, this study will provide a more complete knowledge base on the treatment of metastatic GC by trifluridine/tipiracil, which will contribute to a deeper understanding of trifluridine/tipiracil, and may provide a reliable basis for clinicians.

## Author contributions

Xiaoyan He and Tianyao Zhang equally contributed to the study.

**Conceptualization:** Xiaoyan He, Tianyao Zhang.

**Data curation:** Tianyao Zhang, Xin Zhou.

**Formal analysis:** Lijuan Wu, Yongcan Wu.

**Investigation:** Lijuan Wu, Xin Zhou.

**Methodology:** Yongcan Wu, Xin Zhou.

**Software:** Lijuan Wu, Xin Zhou.

**Supervision:** Xin Zhou.

**Writing – original draft:** Xiaoyan He, Tianyao Zhang.

**Writing – review & editing:** Xiaoyan He, Tianyao Zhang.

## Abbreviations

Abbreviations: 5-FU = 5-fluorouracil, CI = confidence intervals, DNA = deoxyribonucleic acid, ECOG = Eastern Cooperative Oncology Group, GC = gastric cancer, HRs = hazard ratios, OS = overall survival, PFS = progression-free survival, PRISMA-P = Preferred Reporting Items for Systematic Reviews and Meta-Analyses Protocols, RCTs = Randomized controlled trials, RRs = risk ratios, TAS-102 = trifluridine/tipiracil (Lonsurf), TP = thymidine phosphorylase, US = United States.

## References

[1] Shitara K Doi T Dvorkin M. Trifluridine/tipiracil versus placebo in patients with heavily pretreated metastatic gastric cancer (TAGS): a randomised, double-blind, placebo-controlled, phase 3 trial. Lancet Oncol 2018;19:1437–48. 30355453 10.1016/S1470-2045(18)30739-3

[2] Rawla P Barsouk A. Epidemiology of gastric cancer: global trends, risk factors and prevention. Prz Gastroenterol 2019;14:26–38. 30944675 10.5114/pg.2018.80001 PMC6444111

[3] Thrift AP El-Serag HB. Burden of Gastric Cancer. Clin Gastroenterol Hepatol 2020;18:534–42. 31362118 10.1016/j.cgh.2019.07.045 PMC8859863

[4] Digklia A Wagner AD. Advanced gastric cancer: current treatment landscape and future perspectives. World J Gastroenterol 2016;22:2403–14. 26937129 10.3748/wjg.v22.i8.2403 PMC4768187

[5] Salati M Orsi G Smyth E. Gastric cancer: translating novels concepts into clinical practice. Cancer Treat Rev 2019;79:101889. 31445415 10.1016/j.ctrv.2019.101889

[6] Peeters M Cervantes A Moreno Vera S. Trifluridine/tipiracil: an emerging strategy for the management of gastrointestinal cancers. Future Oncol 2018;14:1629–45. 29701076 10.2217/fon-2018-0147

[7] Lee JJ Chu E. Adherence, dosing, and managing toxicities With trifluridine/tipiracil (TAS-102). Clin Colorectal Cancer 2017;16:85–92. 28242161 10.1016/j.clcc.2017.01.003 PMC5743195

[8] Doi T Ohtsu A Yoshino T. Phase I study of TAS-102 treatment in Japanese patients with advanced solid tumours. Br J Cancer 2012;107:429–34. 22735906 10.1038/bjc.2012.274 PMC3405214

[9] Burness CB Duggan ST. Trifluridine/tipiracil: a review in metastatic colorectal cancer. Drugs 2016;76:1393–402. 27568360 10.1007/s40265-016-0633-9

[10] Zaniboni A Bertocchi P Barni S. TAS-102 (Lonsurf) for the treatment of metastatic colorectal cancer. a concise review. Clin Colorectal Cancer 2016;15:292–7. 27431756 10.1016/j.clcc.2016.06.003

[11] Bando H Doi T Muro K. A multicenter phase II study of TAS-102 monotherapy in patients with pre-treated advanced gastric cancer (EPOC1201). Eur J Cancer 2016;62:46–53. 27208903 10.1016/j.ejca.2016.04.009

[12] Moher D Shamseer L Clarke M. Preferred reporting items for systematic review and meta-analysis protocols (PRISMA-P) 2015 statement. Syst Rev 2015;4:1. 25554246 10.1186/2046-4053-4-1 PMC4320440

[13] Edge SBBD Compton CC Fritz AG. AJCC cancer staging manual (7th ed). New York, NY: Springer; 2010.

[14] Eisenhauer EA Therasse P Bogaerts J. New response evaluation criteria in solid tumours: revised RECIST guideline (version 1.1). Eur J Cancer 2009;45:228–47. 19097774 10.1016/j.ejca.2008.10.026

[15] National Institutes of Health, N.C.I. Common Terminology Criteria for Adverse Events v4.0 (CTCAE).2009. https://evs.nci.nih.gov.

[16] Higgins JP Altman DG Gøtzsche PC. The Cochrane Collaboration's tool for assessing risk of bias in randomised trials. BMJ 2011;343:d5928. 22008217 10.1136/bmj.d5928 PMC3196245

[17] Higgins JP, GS. Cochrane Handbook for Systematic Reviews of Interventions Version 5.0.2. The Cochrane Collaboration, 2009. Available from http://handbook.cochrane.org.

[18] Tan Z. Recent advances in the surgical treatment of advanced gastric cancer: a review. Med Sci Monit 2019;25:3537–41. 31080234 10.12659/MSM.916475 PMC6528544

[19] Kawazoe A Shitara K. Trifluridine/tipiracil for the treatment of metastatic gastric cancer. Expert Rev Gastroenterol Hepatol 2020;14:65–70. 31920125 10.1080/17474124.2020.1715209

[20] Biagioni A Skalamera I Peri S. Update on gastric cancer treatments and gene therapies. Cancer Metastasis Rev 2019;38:537–48. 31486976 10.1007/s10555-019-09803-7

[21] Kang C Dhillon S Deeks ED. Trifluridine/tipiracil: a review in metastatic gastric cancer. Drugs 2019;79:1583–90. 31489588 10.1007/s40265-019-01195-w PMC6751145

[22] Jeffers KD. Trifluridine/tipiracil: old drug, new tricks. J Adv Pract Oncol 2016;7:449–53. 29226002 10.6004/jadpro.2016.7.4.7 PMC5679033

[23] Emura T Suzuki N Yamaguchi M. A novel combination antimetabolite, TAS-102, exhibits antitumor activity in FU-resistant human cancer cells through a mechanism involving FTD incorporation in DNA. Int J Oncol 2004;25:571–8. 15289858

[24] Matsuoka K Nakagawa F Kobunai T. Trifluridine/tipiracil overcomes the resistance of human gastric 5-fluorouracil-refractory cells with high thymidylate synthase expression. Oncotarget 2018;9:13438–50. 29568368 10.18632/oncotarget.24412 PMC5862589

[25] Trifluridine/tipiracil for colorectal cancer. Aust Prescr 2018;41:171. 30410217 10.18773/austprescr.2018.053 PMC6202293

[26] Mayer RJ Van Cutsem E Falcone A. Randomized trial of TAS-102 for refractory metastatic colorectal cancer. N Engl J Med 2015;372:1909–19. 25970050 10.1056/NEJMoa1414325

